# Familial Polythelia associated with dental anomalies: a case report

**Published:** 2014-03-30

**Authors:** Gabriel M Fonseca, Mario Cantín

**Affiliations:** 1 Pathology Department, Faculty of Dentistry, Universidad Nacional de Córdoba, Argentina.; 2 Doctoral Program in Morphological Sciences, Faculty of Dentistry, Universidad de La Frontera, Temuco, Chile; 3 Research Center for Biomedical Sciences, Universidad Autónoma de Chile, Temuco, Chile

**Keywords:** Polythelia, dental anomalies, syndrome, inheritance, prevention, cancer

## Abstract

Polythelia has been defined as the presence of supernumerary nipples without accessory glandular tissue. Usually, these growths follow imaginary mammary lines running from the armpits to the groin.

Although the presence of dental anomalies may occasion only a simple cosmetic problem with specific clinical considerations, the association with familial polythelia has been scarcely reported.

This paper reports on a case of polythelia that is associated with dental anomalies in an Argentine family and discusses suggestions for a thorough dental history and medical consultation to prevent possible pathological conditions or potential malignant transformation of mammary tissues.

## Abstract (es)

La politelia ha sido definida como la presencia de pezones supernumerarios sin tejido accesorio glandular; generalmente, éstos siguen las líneas mamarias que discurren imaginariamente desde las axilas hasta la región inguinal.

Si bien la presencia de anomalías dentarias puede originar un simple problema estético con específicas consideraciones clínicas, la asociación con politelia familiar ha sido escasamente informada.

Se presenta un caso de politelia asociada con anomalías dentarias en una familia argentina, y se discuten sugerencias para una cuidadosa anamnesis odontológica y derivación a la consulta médica para prevenir eventuales condiciones patológicas o una potencial transformación maligna de los tejidos mamarios.

## Introduction

Polythelia has been defined as the presence of supernumerary nipples without association with other anatomical glandular structures; normally they follow the path of the mammary line from the armpit to the pubic region 1. It results in the persistence of ectoderm vestiges during the third month of intrauterine development 2 and its frequency varies between 0.2% and 5.6% by sex, ethnicity and geographical area 3 ^,^ 4.

It has been described with different inheritance patterns 5 and is associated with congenital abnormalities in the kidney or the urinary tract^1^. Goldschmidt and Jacobsen have reported a new family syndrome that affects the first pharyngeal arch structures and mammary line 6. However, the presence of dental malformations (usually the reason for the dental visit) associated with familial polythelia is a rare finding and is scarcely described in the literature 7 ^-^ 10. A case of dental anomalies and polythelia in an Argentine family is presented where the association was detected in the dental office during the history taking interview.

## Case presentation

**Figure 1.**
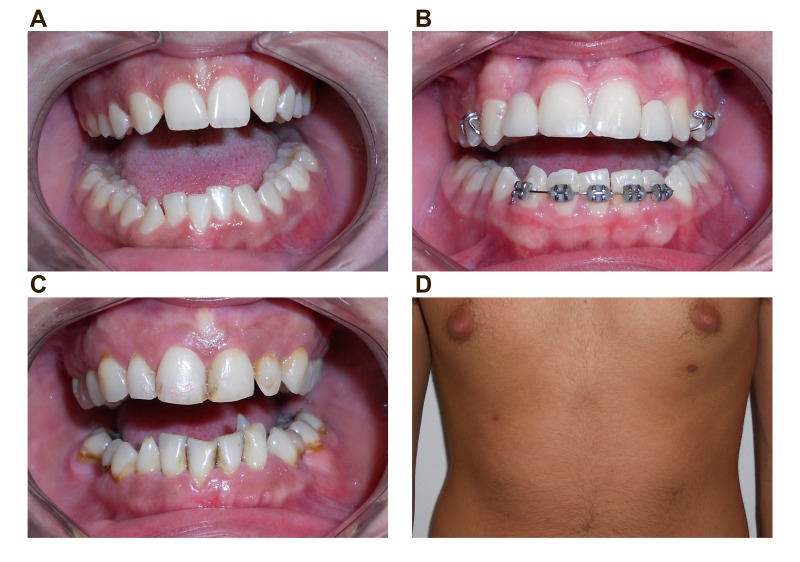
A. Teeth agenesis in the daughter. B. Teeth agenesis and replacement with a removable dental prosthesis in son. C. Dental anomalies in the mother. D. View of supernumerary nipples in the son.

The patient was a 19 year-old female that presented for aesthetic dental consultation for agenesis of both maxillary lateral incisors and a mandibular lateral left incisor Fig. 1A). After a thorough clinical examination and gathering of a medical history, she reported that her brother and mother suffered similar dental anomalies and for this reason the family members were given an appointment. At this appointment it was confirmed that her fifteen year-old brother had agenesis of both maxillar lateral incisors (both had been replaced with a removable prosthesis) and of the mandibular left canine (Fig. 1B). The mother of both was a 46-year-old woman who presented with pronounced lingualization of the mandibular left canine, persistence of the mandibular left second temporary molar (by agenesis of the second left premolar) and conoidism of the maxillar left lateral incisor (Fig. 1C).

All families reported having supernumerary nipples: the women with one on either side of the mammary line, the male with two on the left side and one on the right (Fig. 1D). The individuals noted the absence of renal, neurological or other disorders or malformations other than those reported and were referred for medical follow-up. No other pathological condition was detected. The individuals reported the absence of dental anomalies or polythelia in other family members.

## Discussion

Polythelia represents a typical example of atavism and the word means "many nipples"^11^. It is considered the most frequent malformation of breast tissue, and various forms of genetic transmission have been reported 2. Supernumerary nipples are located on the mammary line and are usually asymptomatic. They are usually unilateral, and its association with renal and urinary tract malformations has already been reported 1 ^,^ 3 ^,^ 5 ^,^ 11.

The genetic transmission of polythelia appears to be heterogeneous and the most common modes are: autosomal dominant with incomplete penetrance and a dominant X-linked chromosome. Each of these modes has demonstrated intrafamilial variability in their clinical manifestations.^11^ There are reports of polythelia being associated with cardiac malformations with pulmonary hypertension, pre-or postnatal overgrowth, dysmorphic facial features, cleft palate, postaxial polydactyly, and a well-established clinical finding is the association with Simpson-Golabi-Behme syndrome 4 ^,^ 8. Goldschmidt and Jacobsen have described a new syndrome as the presence of malformations of the first pharyngeal arch and the mammary line in a family of four generacions^6^. Although the expressiveness of epibulbar lipodermoides was variable, all individuals had polythelia and pre-auricular manifestations. In none of the cases were dental defects or cranial abnormalities identified 7.

Tooth agenesis is the most common anomaly of dental development and may occur as an isolated entity or that composing well-documented syndromes. These clinical situations appear to be due to chromosomal defects or mutations of the genes responsible for organogenesis 12. The association with other genetic abnormalities can occur in the expression of other accessory structures, a situation that was evident in the case presented. Similarly, some form of dental anomalies (such as conoidismo) has been observed in syndromic frames 13.

Although the presence of dental anomalies may suppose a simple cosmetic problem with specific clinical considerations, the scantly referenced associations with polythelia (in syndromes of greater diagnostic complexity 9 ^,^ 10) highlights a topic of undoubted semiological value. When there are no classical syndromic associations, polythelia may be under-diagnosed, especially if the tissue is in proximity to the sweat glands 14. Its exact diagnosis is crucial because a breast carcinoma can be generated in such aberrant areas. Ductal carcinoma has been reported as the most frequent subtype of primary ectopic breast cancer, besides medullary breast cancer, and cystosarcoma phylloides, extramammary Paget's disease and papillary carcinoma 8. We agree with the literature that advises a careful anamnesis and medical consultation for a full investigation into possible pathological conditions or potentially malignant transformation of these accessory tissues 2 ^,^ 4 ^,^ 8.
